# In vivo quantification of quantum dot systemic transport in C57BL/6 hairless mice following skin application post-ultraviolet radiation

**DOI:** 10.1186/s12989-017-0191-7

**Published:** 2017-04-14

**Authors:** Samreen Jatana, Brian C. Palmer, Sarah J. Phelan, Robert Gelein, Lisa A. DeLouise

**Affiliations:** 1 Department of Biomedical Engineering, University of Rochester, Rochester, NY USA; 2 Department of Environmental Medicine, University of Rochester Medical Center, New York, USA; 3 Department of Dermatology, University of Rochester Medical Center, Dermatology and Biomedical Engineering, 601 Elmwood Avenue, Box 697, Rochester, NY 14642 USA

**Keywords:** Quantum dots, Atomic absorption spectroscopy, Distal organ analysis, Skin dendritic cells

## Abstract

**Background:**

Previous work has demonstrated size, surface charge and skin barrier dependent penetration of nanoparticles into the viable layers of mouse skin. The goal of this work was to characterize the tissue distribution and mechanism of transport of nanoparticles beyond skin, with and without Ultraviolet Radiation (UVR) induced skin barrier disruption. Atomic absorption spectroscopy (AAS), flow cytometry and confocal microscopy were used to examine the effect of UVR dose (180 and 360 mJ/cm^2^ UVB) on the skin penetration and systemic distribution of quantum dot (QD) nanoparticles topically applied at different time-points post UVR using a hairless C57BL/6 mouse model.

**Results:**

Results indicate that QDs can penetrate mouse skin, regardless of UVR exposure, as evidenced by the increased cadmium in the local lymph nodes of all QD treated mice. The average % recovery for all treatment groups was 69.68% with ~66.84% of the applied dose recovered from the skin (both epicutaneous and intracutaneous). An average of 0.024% of the applied dose was recovered from the lymph nodes across various treatment groups. When QDs are applied 4 days post UV irradiation, at the peak of the skin barrier defect and LC migration to the local lymph node, there is an increased cellular presence of QD in the lymph node; however, AAS analysis of local lymph nodes display no difference in cadmium levels due to UVR treatment.

**Conclusions:**

Our data suggests that Langerhans cells (LCs) can engulf QDs in skin, but transport to the lymph node may occur by both cellular (dendritic and macrophage) and non-cellular mechanisms. It is interesting that these specific nanoparticles were retained in skin similarly regardless of UVR barrier disruption, but the observed skin immune cell interaction with nanoparticles suggest a potential for immunomodulation, which we are currently examining in a murine model of skin allergy.

**Electronic supplementary material:**

The online version of this article (doi:10.1186/s12989-017-0191-7) contains supplementary material, which is available to authorized users.

## Background

The commercial use of engineered nanomaterials is rapidly expanding in the fields of targeted therapeutics, biomedical diagnostics, cosmeceuticals and electronics [1]. Some nanoparticles (NP) used in commercial and research applications include quantum dots (QD), carbon nanotubes (CNT), fullerenes, metals (Au, Ag), metal oxides (TiO_2_, ZnO, SiO_2_) and lipophilic nanoparticles (liposomes) [2–7]. The global market value for NP in biotechnology, drug development and delivery is expected to reach 53.5B US dollars by 2017 with personal care products containing NPs accounting for the largest share in the nanotechnology market today [8].

The unique physical, optical and tactile properties of NPs make them ideal for use in topical skin applications [8]. By far, the largest application is formulation in ultraviolet radiation (UVR) protective sunscreens and daily use skin care products that contain zinc oxide (ZnO) and/or titanium dioxide (TiO_2_) NPs. This has driven considerable research to investigate the interaction of metal oxide NPs with skin using different animal and human models [9–12] however, little is understood about the interaction of NPs with UVR exposed skin [13]. UVR skin exposure has been shown to cause production of reactive oxygen species (ROS), formation of cyclobutane pyrimidine dimers (CPD) and release of cytokines that lead to time dependent epidermal damage, erythema and immunosuppression [14–18]. UVR exposure induces keratinocyte hyper-proliferation that leads to a thickened epidermis, a malformed stratum corneum and defective skin barrier that peaks 3 to 4 days post UVR as measured by transepidermal water loss (TEWL) [19]. Although both UVA and UVB skin exposure have been directly linked to sunburn, photoaging, and carcinogenesis, the consequence of frequent use of nano-enabled products on UVR induced barrier defective skin is not well characterized [20, 21]. It is anticipated that NP skin penetration would be more likely after UVR exposure, and thus it is important to study this from a toxicological perspective. If NPs penetrate through the stratum corneum into the viable epidermis they then have the potential to be transported to distal organs either by cellular uptake or through the lymphatic circulation and blood stream [19, 22].

Studying NP skin penetration, local cellular environment interaction, and transport to distal organs are major challenges in the nanotoxicology field due to difficulties in tracking and quantifying many types of nanoparticles. Analytical techniques commonly used to track the absorption and distribution of NP in tissues include confocal microscopy and flow cytometry which rely on fluorescence detection. Hence, fluorescent semiconductor CdSe/ZnS core/shell (cadmium selenide core, zinc sulphide shell) quantum dot (QD) nanocrystals have been widely used as a model NP for nanotoxicology. Of note, there is primary interest in understanding QD skin interactions due to their increasing importance in the biomedical, electronics, optics and energy fields that present an increased risk for QD body exposure [23–25]. In addition to their size dependent optical properties, QDs are also advantageous in that their surface chemistry can be modified to alter charge and render them soluble in aqueous application vehicles. Mass spectrometry and atomic absorption spectroscopy (AAS) are also used to detect elemental cadmium (Cd) in tissues providing indirect evidence for the presence of QD particles [19, 22, 26–28]. These techniques are more quantitative and have higher detection sensitivity than fluorescent microscopy, which suffers from high background signal in tissue specimens. Taking advantage of these attributes, Liu et al. used inductively coupled plasma mass spectrometry (ICP-MS) to examine the in vivo breakdown of QDs (cadmium-telluride core) in mice by tracking the ratio of both metal ions systemically over time after a single intravenous injection [29]. This study reported evidence for some physiological breakdown of QDs into their component metal ions over a 28-day period with both cadmium and telluride ions mainly accumulating in the liver [29]. In another study, polyethylene glycol (PEG) coated QDs (CdSe core, CdS shell) were intradermally injected in mice to quantify the biodistribution in distal organs using ICP-MS. This study also found that over a period of 24 h the QDs accumulated in the draining lymph nodes and other major organs including the liver and kidney (kidney filtration size 5-6 nm) [30].

While intradermal and intravenous injections are the most common routes of QD delivery into animals only a few studies have attempted to track QD systemic distribution in mice following a topical application [19, 31]. Examination of the topical delivery route is important since consumers are more likely to apply nano-enabled cosmetics to their skin [9]. Gopee et al., found that polyethylene glycol coated QDs (37 nm hydrodynamic diameter) topically applied on intact skin of SKH-1 hairless mice were below the levels of detection in the sentinel organs analyzed using ICP-MS [31]. Elevated Cd levels were detected in dermabraded skin at 24 and 48 h post application [31]. In our previous study of QD penetration through UVR (360 mJ/cm^2^ UVB) exposed skin using AAS we observed a statistically significant increase in liver Cd levels when the QDs were applied topically to the back skin on day 4 post irradiation at the peak of barrier defect [19]. Unexpectedly, Cd was detected in the lymph nodes of control animals (no UVR) in this previous study, but the mechanism of transport was unclear [19] and we did not attempt to quantify QD presence in other organs or at other time points post UVR exposure or to account for the total applied dose.

In the present study we used AAS to quantify the QD (CdSe core, ZnS shell) distribution via Cd concentration in skin (both penetrated and non-penetrated levels), pooled skin draining lymph nodes (axillary, brachial, and cervical), liver, spleen, intestine (small and large), and feces. The purpose of the study is twofold: 1) to quantitatively track QD penetration through skin to distal organs when topically applied at different time points post-UV irradiation to establish trends with the development of the UVR induced skin barrier disruption, and 2) to examine the transport mechanisms of QDs to the lymph nodes after QD exposure on control or UV irradiated skin using flow cytometry.

## Methods

### Quantum Dot functionalization and measurement

QD605 quantum dots with a cadmium/selenide core and a zinc/sulphide shell, capped with octadecylamine, and suspended in a toluene solvent, were purchased (#CZ600 NN-Labs, Fayetteville, Arkansas) and chemically modified in-house to make the QDs negatively charged and water-soluble. Hydrophilic ligand coating was added on the QD surface to enable physiological use in an in vivo model. We and others have shown that ligand composition and surface charge can affect QD cell uptake, cytosolic trafficking and cytotoxicity. For this study we chose glutathione (GSH), which is a superior coating based our studies of colloidal stability, quantum yield and minimal keratinocyte cytotoxicity [26].

To functionalize the QDs with GSH (Calbiochem, CAS 70-18-8), 300 μl of the QD stock solution (10 mg/ml) was added to 1700 μl of a 1:1 methanol: acetone mixture and centrifuged at 14,000 RPM for 5 min. The supernatant was removed and the QD pellet was dried via nitrogen flow. The QD pellet was suspended in 300 μl of dried tetrahydrofuran immediately after drying. In a separate glass vial, 30 mg GSH was dissolved in 1 mL of methanol and the pH was adjusted to 11 by adding tetramethylammonium hydroxide (Sigma-Aldrich, #T7505). The GSH mixture was stirred under constant heat at 60^o^ C, and slowly the QD suspension was added drop wise. The mixture was stirred at a constant 60^o^ C for an additional 2 h then the heat was removed and the mixture was stirred overnight. The following day, the mixture was transferred evenly to two centrifuge tubes filled with 1.5 mL each of diethyl ether. Once vortexed, the ether and QD mixture was centrifuged at 14,000 RPM for 5 min. The supernatant was removed and the pellet was dried under nitrogen flow. The two QD pellets were dissolved in 100 μl of 0.1 N NaOH, demonstrating the water solubility of the newly coated particles, and transferred to a dialysis tube for removal of unbound GSH (5-kD cut-off DispoDialyzer filter, Harvard Apparatus Inc., Holliston, Massachusetts). The dialysis tube was placed in a light protected 50 mL vial filled with DI water (pH = 6.7); and then placed on a plate rocker for 2 days at 4 ° C, with the DI water replaced once after the first 24 h of incubation.

To determine the concentration of the functionalized QD mixture we placed 2 μl of undiluted sample onto a NanoDrop spectrophotometer and measured the peak absorbance at 585 nm. Beer-Lambert’s law was used to calculate the concentration of the QDs [26]. The average hydrodynamic diameter (57.92 nm), zeta potential (-57.1 mV) and polydispersity (0.459) were measured using the Malvern Zetasizer Nano ZN in deionized water (Malvern Instruments Ltd., Worcestershire, United Kingdom) at pH = 6.5.

### Animal treatments: UVR dosing protocol

Animal experiments were approved by the University Committee on Animal Resources (UCAR) at the University of Rochester Medical Center (#100360/2010-024). All mice used in this study are hairless SKH-1 mice backcrossed 7 generations into a C57BL/6 mouse background. The SKH-1 mouse contains a mutation in the *hairless* (*Hr*) gene that causes alopecia to develop after the first hair follicle cycle. This phenotype is preferred for UVR exposures, since the use of other breeds necessitates hair removal, which may cause a barrier defect in the epidermis. Also C57BL/6 hairless mice retain their hair follicles, which is a possible route for NP accumulation and penetration through skin. Mice were either male or female with ages that range from 5 to 8 months old. The mice were housed in standard cages, up to four mice per cage, with access to food and water ad libitum. However, after UVR and QD exposure, the mice were housed individually to prevent interactions that could alter the skin barrier and QD penetration.

Ultraviolet light has three distinct wavelength ranges: UVA (400-315 nm) is the long wave form, UVB (315-280 nm) is the medium wave form that can be most damaging, and UVC (280-100 nm) is the shortest wave form that is absorbed by the ozone layer. To expose the mice, we used a UVA Sun 340 lamp that emits in both the UVA and UVB wavelengths closely resembling the damaging portion of the UVR spectrum produced by the sun (300-400 nm) [32]. The 180 mJ/cm^2^ and 360 mJ/cm^2^ dose was tuned to the UVB range by using a calibrated IL1700 light meter (International Light) with a SED 240 probe, and the exposure time was calculated using the measured flux value (J/cm^2^-sec). An equivalent exposure to UVB of 180 mJ/cm^2^ would be approximately achieved by exposure to the sun at noon in mid-July in Rochester, NY for approximately 11 min. To expose the mice to UVR, they were housed separately, without bedding, in open top cages 15 in. away from the lamp [22].

To examine the effect of UVR on Langerhans cell (LC) density, mice were irradiated using a dose calibrated on UVB and then euthanized at the following time points: 0 h, 1, 4, 7, 9 and 14 days after irradiation. Skin was harvested from the back (5 cm^2^ area) using a stainless steel surgical blade (Miltex, Inc.) and stored at -80 °C for immunofluorescence (IF) analysis.

### Quantum Dot application and AAS protocol

**Fig. 1:**
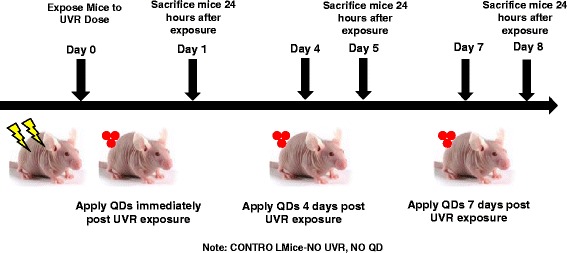
Schematic of the UV radiation and quantum dot (QD) exposure protocol using C57BL/6 hairless mice. For each treatment; mouse skin, draining lymph nodes, liver, intestine, and feces were collected for AAS analysis of cadmium (Cd) concentration. PDMS strips used during QD application, and gauze used to wipe the skin surface were also analyzed for Cd. Mice that received no UVR and no QD treatment served as controls

Mice (*n* = 4) were treated with QDs 0, 4, or 7 days post UVR exposure for 24 h (Fig. 1). There were also two control groups: one group was not exposed to UVR radiation or QDs and the other group was exposed to only QDs (no UVR) (*n* = 4). QDs (~5.72 × 10^−11^ mol, 2.24 μl) were mixed in 0.05 g of Eucerin® Dry Skin Therapy plus Intensive Repair (Beiersdorf Inc.) that was shown to enhance the skin penetration of QDs due to the high concentration of alphahydroxy acids; it represents a common commercial skin lotion widely used and mimics the skin application of a sunscreen [33]. The QD lotion mixture was applied to the back of each mouse with a PDMS (polydimethylsiloxane, 1.5 cm^2^ in area) applicator. The PDMS was made by mixing 25 mL PDMS elastomer base and 2.5 g of elastomer curing agent, and then pouring the mixture into a mold and curing overnight. The PDMS application strip and the pipette used to aliquot the QDs were both analyzed by AAS to determine QD loss via the application method.

The experimental set-up mimics the application of sunscreens containing nanoparticles after UV irradiation in humans, so rather than using a non-occlusive dressing, the mice were fitted with an Elizabethan collar (Braintree Scientific, Massachusetts) and housed in bedding free cages for the duration of QD exposure to limit QDs lost to ingestion by grooming. The mice were held by their tails and a fresh PDMS strip was used to gently apply the QD lotion mixture on their back. The QDs were applied to the dorsal side of the mouse from head to tail, and the QD mixture was rubbed gently to distribute evenly on the skin to cover a surface area of ~10 cm^2^. The pipette tip and the PDMS applicator were collected for AAS analysis. The following day, 24 h after QD application, the mice were sacrificed by CO_2_asphyxiation and cervical dislocation. Care was taken not to disturb the back of the mouse where a large proportion of QDs remained. After euthanasia, QDs on the skin surface were gently wiped off using gauze soaked in 1X phosphate buffered saline (PBS) and this was analyzed by AAS to quantify the residual QD skin concentration (epicutaneous).

The procedure for removal of the mouse organs and preparation for graphite furnace AAS has been previously described [19]. The organs were harvested using dedicated dissection instruments to avoid contamination between treatment groups. The tissues and other samples were directly placed in pre-weighed 50 ml tubes and the sample weights were recorded before ashing with nitric acid (Baseline, SeaStar Chemicals Inc.). Full thickness skin (epidermis and dermis) was harvested from the mouse dorsum (neck to tail) using dissection scissors and a scalpel. Cadmium levels were quantified in each sample by comparison to reference standards. The limit of detection (LOD = 0.004 ng/ml) and the limit of quantification (LOQ = 0.013 ng/ml) were established as previously described [19]. In this study, the gauze (epicutaneous), skin (penetrated skin dose in the epidermis and dermis-intracutaneous), lymph nodes (axillary, brachial and cervical), liver, intestines (duodenum to the last fecal pellet of the large intestine), excreted feces, spleen and PDMS applicators were analyzed using AAS. The lymph nodes were pooled for analysis. A preliminary analysis showed that the Cd levels were below LOD when lymph nodes were analyzed individually. Critical parameters to control in NP topical skin exposure studies are the area of application and minimizing grooming to prevent ingestion of the NP for the duration of the exposure. In this work extreme care was taken to prevent grooming by placing collars on the mice. Nonetheless, we specifically analyzed the feces for elemental Cd in the UVR treated mice (collared) and compared it to both mice treated with oral gavage and mice treated topically with no collar. Here, we limited the topical application of the QDs to a small area between the scapulae of the mice where they are less able to groom. This is thought to reduce the risk of oral ingestion through grooming with and without use of the collar.

To prove this we performed an oral gavage as a control where mice were dosed with 50 uL of 2.5x10^−11^ mol of QDs (50% of the topical dose) and samples were taken 24 h later (*N* = 4). We were concerned about acute Cd toxicity in the oral gavage study so we lowered the dose compared to the topical exposures. A significantly higher amount of Cd was detected in the feces of mice with the oral gavage treatment compared to UVR treated mice which was expected since ingestion was the only exposure route (Additional file 1: Figure S1). Mice (collared and uncollared) were treated to an equivalent topical dose for 24 h. Interestingly, the Cd levels detected in the feces of mice with no collar were significantly higher than those of the collared UVR treated mice (*p* < 0.05) and they were as high as the oral gavage mice that received half the dose applied to skin indicating the need to use collars to prevent QD ingestion from grooming. Langerhans cell migration kinetics: Immunofluorescence and confocal imaging.

For immunofluorescence, the skin was removed from the -80 °C storage and allowed to thaw at room temperature. For each experimental set-up, skin samples of about ~1 cm^2^ were cut and surgical blade was used to remove the subcutaneous fat and thin the dermal layer leaving the epidermis intact. The samples were then stained for LCs (anti-langerin CD207 conjugated with Alexa 488, eBioscience, Cat No: 53-2073-82). The skin sample was placed in methanol at -20 °C for 15 min for fixing and then blocked at room temperature using 2% bovine serum albumin (BSA, HyClone, Cat No-SH30574.01) in phosphate buffered saline (1X PBS). Next, 2 μl Fc block (anti-mouse CD16/CD32, eBioscience, Cat No-14-0161-82) was added to 100 μl 2% BSA solution and the skin was immersed in it for 40 min at 4 °C to block all the non-specific Fc gamma III and gamma II receptors. Next, 2 μl CD207 anti-langerin antibody was mixed in 50 μl 2% BSA solution. Skin was immersed in the solution containing CD207 antibody overnight at 4 °C. After the overnight incubation, skin was washed thoroughly (x2) in distilled water and the stained samples were placed in a glass bottom microwell dishes (MatTek Corporation) with the epidermis facing down. The samples were coated with Mowiol (embedding medium, Sigma Aldrich #81381) and flattened using a cover slip for confocal imaging using the FV1000 Olympus Laser Scanning Confocal Microscope. The number densities of the positively stained LCs in the images captured on the microscope were quantified using image J software (NIH version 1.45).

### Sample preparation for flow cytometric analysis

The UVR irradiation and QD exposure protocols were similar to that described under the AAS section. Lymph nodes were extracted from mice in each treatment group and placed on ice. The lymph nodes were divided into 3 groups: axillary, brachial and cervical. RPMI media (Sigma-Aldrich) with 1 M HEPES and 12.5 ml fetal bovine serum (FBS) was used for processing. Lymph node digestion was performed in 1 mg/ml of collagenase type II (125 U/ml Gibco, Cat No: 17101-015) and 50 μl of total DNase (30 mg/ml, Roche, Cat No: 10-104-159001) added to 50 ml RPMI. The lymph nodes were crushed using frosted slides into a petri dish containing the RPMI media. The slides and petri dish were washed with additional 5 ml of media and transferred to 15 ml tubes. The tubes were incubated at 37 °C for 25 min with occasional agitation. After the incubation period, the digestion was quenched using 5 ml RPMI media and the cells were spun down on a centrifuge (1600 rpm) and 5 ml red blood cell lysis buffer was added to each tube (Eppendorf, Centrifuge 5417C). The cells were incubated on ice for 5 min with occasional agitation. The digestion was quenched using 5 ml RPMI media, the cells were spun down (1600 rpm) and filtered to remove tissue debris. The cell count was determined using trypan blue and automatic cell counter (Bio-Rad). Cells (2 × 10^6^ per sample) were transferred into 1.5 ml Eppendorf tubes for antibody cell staining. After incubating in Fc blocking buffer, the cells were washed and re-suspended in 100 μl cell staining buffer containing the following antibodies: CD207 (Alexa Fluor 488, eBioscience, Cat No: 53-2073-82), MHCII (eFluor 450, eBioscience, Cat No: 48-5321-82), CD19 (PE, eBioscience, Cat No: 12-0193-82), CD11b (PE-Cy5, eBioscience, Cat No: 15-0112-82) and F4/80 (PE-Cy7, eBioscience, Cat No: 25-4801-82). Compensation controls (including an unstained sample) and fluorescence minus one (FMO) controls were added for each antibody including QD605. The antibodies were used in the following concentrations (per 100 μl of the buffer): CD207 (2 μl), MHCII (2 μl), CD19 (0.6 μl), CD11b (1 μl), F4/80 (2 μl) and QD605 (2 μl). QD605 was added only to the compensation and FMO controls. The cells were treated with Fc block (anti-mouse CD16/CD32, eBioscience, Cat No-14-0161-82) for 15 min at 4 °C to prevent nonspecific antibody binding. Next, the specific antibodies were added to each sample and the tubes were incubated on a rocker at 4 °C for 20 min. The samples were washed using the cell staining buffer and spun down. The supernatant was removed and the cells were fixed using 2% paraformaldehyde (Boston BioProducts, Cat No: BM-155) at 4 °C for 15 min. The cells were washed, spun down and re-suspended in 300 μl phosphate buffered saline (1X PBS) for flow cytometry. Data were collected using 18-color LSRII flow cytometer (BD Biosciences) at the flow rate of 35 μl/min.

### Data and statistical analyses

Power analysis was conducted for the number of animals required for the AAS studies and the LC kinetics study (1-β > 0.95). A total of 4 animals per treatment group were used for AAS analysis (*N* = 4). Three animals per treatment group were used for the LC migration studies and flow cytometry analysis (*N* = 3-4). For flow data analysis, results from 3 to 4 experiments with approximately a million cells per treatment group were concatenated. All AAS data is presented as either percent Cd recovered (Tissue Cd concentration/Sham Cd concentration) or as Cd (ng) per total tissue weight (g). All statistical analyses were run with JMP Pro v 12.1.0 (SAS Institute Inc., Cary, NC). A Kruskal-Wallis test and subsequent post-hoc Mann-Whitney analysis was performed when appropriate data with *p*-values <0.05 were considered significant. The confocal microscopy and flow cytometry data are presented as total cells per skin area and total cells per sample, respectively. Appropriate compensation using standard beads (BD Compensation Beads #552843) and fluorescence minus one (FMO) controls were added to each panel for measurement on the 18-color LSR flow cytometer. Data was analyzed using FlowJo (8.8.7). The gating strategy has been shown in Additional file 1: Figure S2. The statistical tests used for each experiment are mentioned under each figure legend in detail.

## Results

### Cadmium (Cd) tissue distribution analysis by atomic absorption spectroscopy (AAS)

**Fig. 2:**
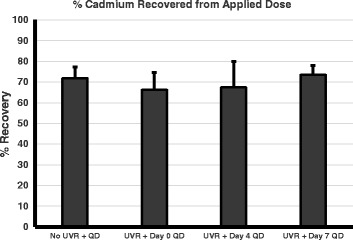
Total QD cadmium recovered in Atomic Absorption Spectroscopy (AAS) quantitative analysis of QD skin penetration. AAS analysis of Cd from QDs measured in the skin (intracutaneous), remaining on the skin wiped with gauze (epicutaneous) and all organ samples compared to the total applied. The average dose topically applied to each mouse was 10.96 +/- 1.21 μg Cd. The total Cd recovered for each treatment ranged from 66.3 to 73.6% of total applied dose. The graph represents the mean +/- standard error (SEM) of the total recovery percentage, *N* = 4. After a Kruskal-wallis test, no significant differences were found between the treatment groups for percentage of total cadmium recovered

**Fig. 3:**
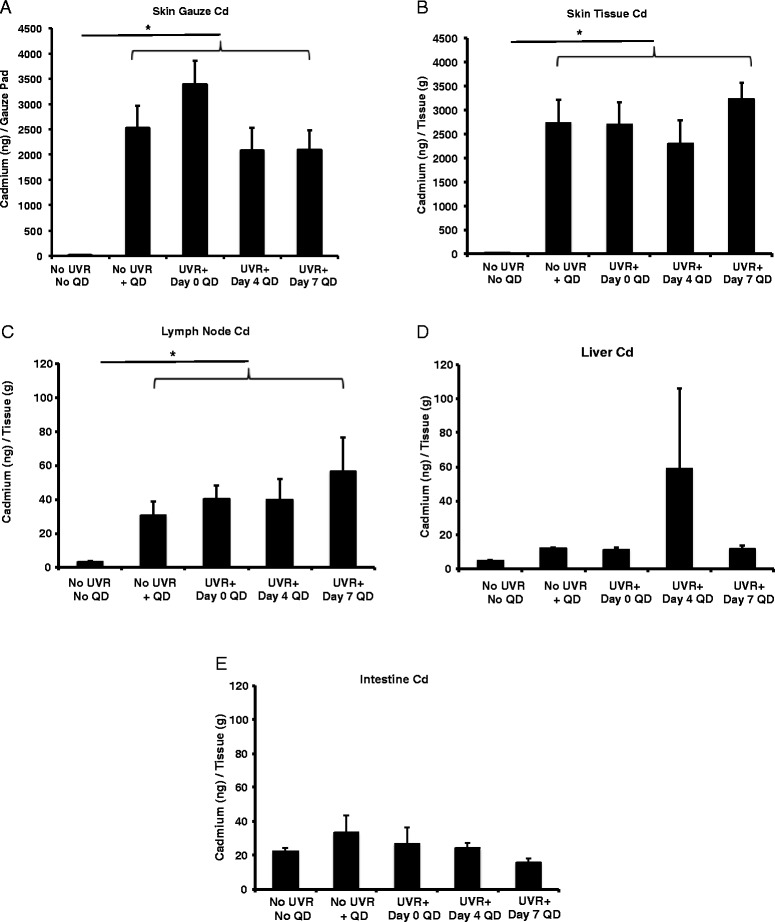
Atomic Absorption Spectroscopy (AAS) quantitative analysis of QD cadmium in organs. Mice were topically exposed to QDs on 0, 4, or 7 days post 180 mJ/cm^2^ UVB radiation exposure for 24 h. The concentration of cadmium (ng/g tissue) was then measured in various organs including the (**a**) gauze (epicutaneous), (**b**) skin (intracutaneous), (**c**) draining lymph nodes, (**d**) liver and (**e**) intestine. The graphs represent the mean +/- standard error (SEM), *N* = 4. After Kruskal-Wallis analysis (*p < 0.05), there are no significant differences between any QD treated groups; however, all QD treated skin tissue Cd groups had significantly higher Cd compared to no treatment control (No QD, No UVR)

Studies were performed to investigate whether the UVR induced inside-out barrier defect as measured by Transepidermal Water Loss (TEWL), correlates with the outside-in penetration of QDs. In prior work, we examined the TEWL values post UVR (90, 180, 270 and 360 mJ/cm^2^ UVB) irradiation from day 0 to day 7 and found that TEWL values peaked days 3–4 in UVR treated mice compared to control [19]. In this study, we treated mice with QDs for 24 h topically applied either on day 0, 4 or 7 post-UVR irradiation (180 mJ/cm^2^ UVB light); day 4 being the peak of barrier defect post UV irradiation. This UVB dose was selected as it induces a significant barrier defect without causing obvious skin erythema [19]. We also included a control group that remained untreated (no QD and no UVR) and a group that was treated only with the QD (no UVR). The mice were euthanized 24 h post-treatment, and the Cd concentration in the skin (epicutaneous and intracutaneous), lymph nodes, intestine, liver, spleen and feces were analyzed by AAS. The maximum Cd dose was 13.74 ± 1.28 μg Cd, measured by AAS analysis of the PDMS applicators loaded with the QD lotion mixture. Accounting for residual QDs left on the PDMS applicators after treating each mouse, the total Cd mouse exposure was 10.96 ± 1.21 μg Cd. The levels of Cd (μg) in all mouse tissues were summed for each treatment group and averaged, which equaled 7.63 ± 1.77 μg Cd. Therefore, the average percent recovered for all QD treatments was 69.68% (Fig. 2). Some Cd could be lost in tissues not analyzed or to accidental transfer to the Elizabethan collar or cage; however, a percent recovery of ~70% of the total Cd applied is exceptional for whole animal distribution analyses and consistent with previous reports [30]. It is noteworthy that the majority of the recovered Cd was from the skin (66.84% of applied dose); both from the epicutaneous (dose wiped off with gauze, skin gauze Cd, Fig. 3a) and the intracutaneous dose remaining in skin (skin tissue Cd, Fig. 3b). The skin tissue Cd (intracutaneous) represents those QDs that have either traversed into or beyond the stratum corneum into the epidermis and dermis or trapped in hair follicles and/or skin furrows. The data indicates that QDs are retained in skin independent of time post UV exposure, since all QD treatments contain significantly more Cd in the skin compared to no treatment control (*p* < 0.05), and there are no significant differences between the UVR exposure groups (Fig. 3b). Interestingly, the No UVR + QD group had similar levels of Cd compared to the UVR treated groups.

This differs from previous work that reported NP penetration through skin occurs in a barrier defect dependent manner [34–37]. TEWL values are highest on day 4 post-UVR exposure corresponding to barrier impairment; however, our AAS results are unable to detect differences in QD penetration (Cd levels) between days 0, day 4 or day 7 post-UVR treated skin. It is plausible the vehicle (Eucerin® lotion) used for the study facilitated QD collection in the stratum corneum, skin folds and follicles creating a significant background signal that masked the effects of UVB exposure and time. Future studies can be designed to employ tape stripping as a means to differentiate QDs trapped in the stratum corneum from those in deeper skin layers and follicles.

The levels of Cd detected in the lymph nodes, liver, intestine/feces made up a small percentage of the total applied dose, 0.024, 0.41, and 2.51% respectively. However, analysis of the lymph nodes (axillary, brachial, cervical and inguinal) and liver suggest that some QDs are able to enter into systemic circulation. The level of Cd in the lymph nodes was higher in all treatments, compared to the no QD treatment control and there were no significant differences between the UVR treatments (Fig. 3c). There also were no significant differences observed in the Cd values obtained for the liver compared to no treatment control (Fig. 3d). The main excretory pathway for QDs is biliary through the liver and intestine, since they are too large to be significantly excreted by the kidneys [38]. There was no significant increase in Cd in the intestine compared to control (Fig. 3e), most likely due to the limited 24 h time frame of the experiment. The small variation in Cd levels in the liver and the intestine may be accounted for by possible differences in dietary Cd intake [39]. Cd levels detected in the spleen were low and no significant differences were detected between the treatment groups and the control (Additional file 1: Figure S3).

**Fig. 4:**
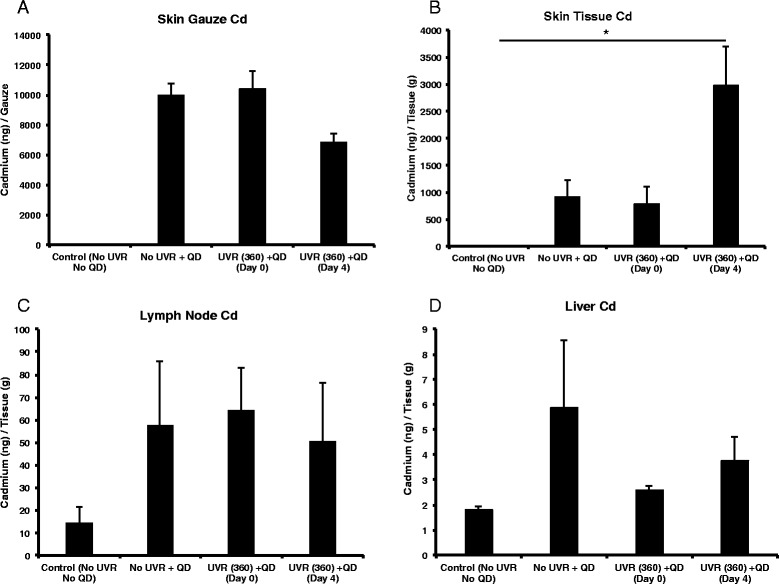
Atomic Absorption Spectroscopy (AAS) analysis data from various organs (360 mJ/cm^2^ UVR dose). The concentration of cadmium (ng/g tissue) found in the gauze (epicutaneous) (**a**), skin (intracutaneous) (**b**), draining lymph nodes (**c**), and liver (**d**). The graphs represent the mean +/- standard error (SEM), *N* = 4. The results were analyzed using a one-way ANOVA (skin and skin gauze) or Kruskal-Wallis (lymph nodes and liver) analysis. The following values were measured in the controls: No UVR + No QD, Mean +/- SD (*n* = 4), Skin = 2.2 +/- 0.96 ng/g, Lymph nodes = 14.55 +/- 6.85 ng/g, Liver = 1.79 +/- 0.14 ng/g. *p < 0.05

Mice were also irradiated using a dose of 360 mJ/cm^2^ and the QDs were applied day 0 and day 4 post-irradiation. Results (Fig. 4) were similar to the 180 mJ/cm^2^ study with the notable exception of a significantly higher level of Cd detected in the skin tissue (intracutaneous) over the control for the day 4 treatment group which suggests QDs accumulation in skin depends on the magnitude of the barrier defect as measured by TEWL.

Since Cd was detected in the lymph nodes, we next examined the transport mechanism. Transport of QDs could possibly occur via skin dendritic cells or non-cellular via the blood stream and lymphatics. The presence of LCs in the skin is dependent on UVR exposure and LCs are known to survey the skin for foreign bodies including NP, so we examined the role of LCs and other immune cells in this process.

### Langerhans cell (LC) migration kinetics

**Fig. 5:**
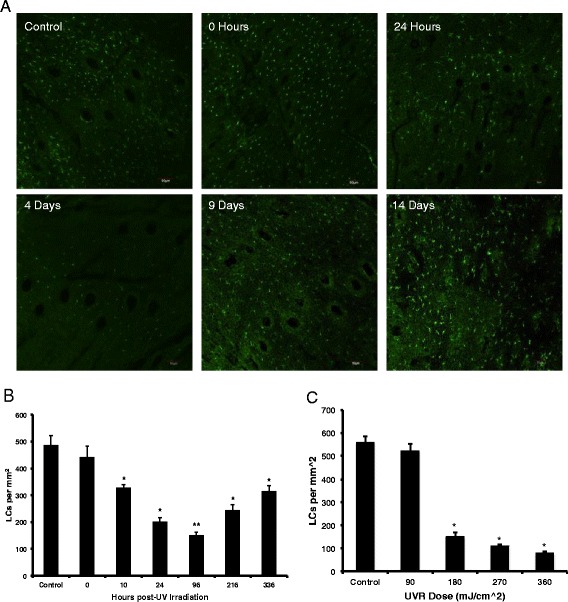
Langerhans cells (LC) migration kinetics. **a** The Langerhans cell (LC) migration was quantified by confocal microscopy of CD207 (langerin marker, Alexa 488-shown in green) stained hairless mouse epidermis measured from 0 h to 14 days after exposure to 180 mJ/cm^2^ UVB radiation, Scale Bar = 10 μm. **b** The bar chart represents the number of LCs per epidermal area over time quantified using ImageJ software. The graph represents the mean +/- standard error (SEM), *N* = 3, *n* = 3 (three regions analyzed for each epidermal sheet imaged). *p < 0.05, **p < 0.0001, 2-Talied *t*-Test, unpaired with unequal variances with respect to control. **c** LC migrations kinetics with respect to to UVR dose response at day 4 post-irradiation. *p < 0.05, 2-Tailed Students *t*-Test with unequal variance with respect to control

LCs are UVR sensitive dendritic cells present in the epidermis. They play an important role in developing immune tolerance to UVR, a phenomenon known as UVR induced immunosuppression, which protects against the development of sun allergies [40–43]. Studies show that following UVR exposure LCs migrate to the local skin draining lymph nodes with dead skin cell debris and present the self-antigen to naïve T cells [44]. This leads to the generation of UVR-induced regulatory cells T cells [45, 46]. Here, we examined the role of skin LCs in the transport of QDs to the lymph nodes. First, we mapped the kinetics of LC migration from the skin post-UVR irradiation by quantifying LC presence in back skin. Skin was obtained from mice at different time-points post-UVR treatments and stained for LCs using anti-langerin Alexa 488 (Fig. 5a). We observed a decrease in the LC population in the skin epidermis following UVR exposure of 180 mJ/cm^2^, with a significant decrease starting at 10 h post-irradiation (Fig. 5b), *p* < 0.05. The LC count was the lowest on day 4 post-irradiation (~70% reduction compared to control, *p* < 0.0001), and the LCs start repopulating the skin on day 9 (Fig. 5b). Results also show that the % reduction of LCs in the skin 4 days post UVR depends on the UVB dose (Fig. 5c). It is of interest to note that when the LC density in skin is the lowest (day 4 post-UVR), the skin barrier defect is the highest as measured by TEWL [19]. Hence, this suggests that if QDs are taken up LCs in skin and cellular transport is an important mechanism, there should be a dependence of QD presence in the lymph node on the time post-UVR exposure that the QDs are applied to skin. To examine if immune cells in the skin are important in QD LN transport we analyzed tissue histology sections and conducted flow cytometry.

### Immunofluorescence and flow cytometry analysis

**Fig. 6:**
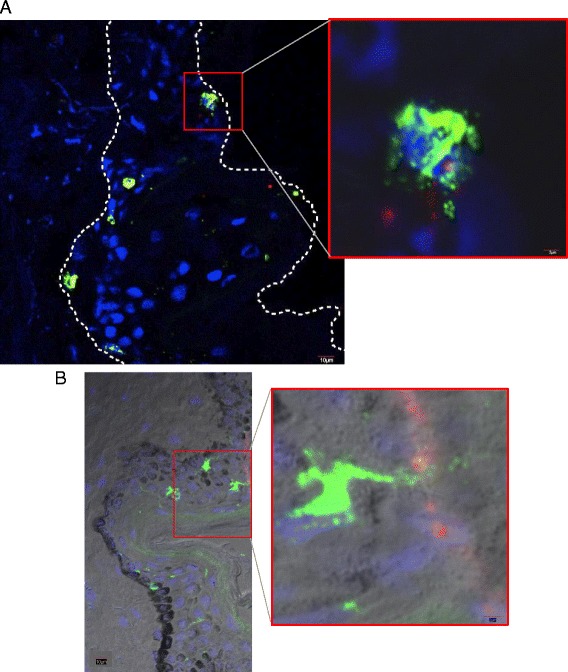
Langerhans cells co-localized with QD in the skin epidermis. Skin cryosections were imaged using the confocal laser-scanning microscope. **a** Immunofluorescence (IF) image showing a Langerhans cell co-localized with a QD cluster near the stratum corneum. **b** IF image superimposed with bright field image showing Langerhans cells in the stratum corneum. The inset shows a LC dendrite extending towards QD clusters present in the epidermis. Scale bar = 10 μm (low magnification image, Scale bar = 2 μm (high magnification image). *Green*-Alexa 488 anti-langerin (LCs), *Blue*-DAPI and *Red*-QD 605

QDs were topically applied on the backs of mice and skin sections were analyzed for QD co-localization with LCs in the epidermis using confocal laser-scanning microscopy. We observed possible uptake of QDs by LCs in some regions of the epidermis near the stratum corneum (Fig. 6, Additional file 2: Video File S4). To develop the flow cytometry protocol we first injected QDs into the dorsal flank of the mice. Imaging data was obtained from this positive control (QDs intradermal injection). Results showed a high co-localization of QDs with LCs in the skin (Pearson’s coefficient = 0.77) (Additional file 1: Figure S5). Cryo-sections also revealed a high presence of QDs in the lymph nodes (Additional file 1: Figure S5). It is important to note that flow cytometry reports events positive for QD605 only when a QD is associated with a cell. It does not quantify nonfluorescent, degraded QDs or non-cellular associated free QDs.



**Additional file 2: Video File S4.** Z-stacks acquired using confocal microscopy. (MOV 506 kb)

**Fig. 7:**
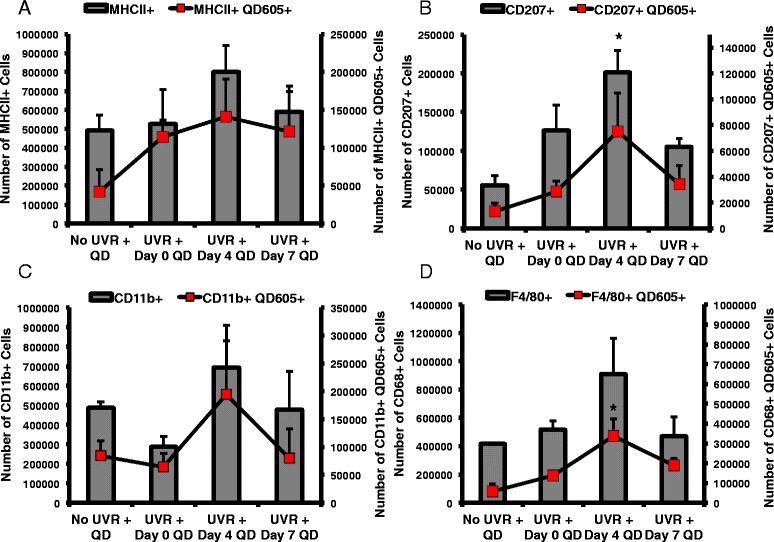
Flow cytometric analysis of leukocytes in the draining lymph nodes of mice. Mice were treated with QDs 0, 4, or 7 days post 180 mJ/cm^2^ UVB radiation exposure for 24 h and the lymph nodes were processed for analysis. Cells positive for the QD605 marker are co-localized with an intact QD. Leukocytes were sorted into general antigen presenting cells (MHCII+) (**a**), Langerhans cells (CD207+) (**b**), monocytes (CD11b+) (**c**), and macrophages (F4/80+) (**d**). The total, isolated leukocyte populations are represented by the grey bars, and the isolated leukocyte/QD605+ double positive cells are represented by the *red squares*. The total events collected in each group were 2 × 10^6^. The graphs represent the mean +/- standard error, *N* = 3. The statistics are based on one-way ANOVA with post hoc Tukey tests, except for the data with unequal variance (F4/80 + QD605+, CD11b+) for which a Kruskal-Wallis test was used (*p < 0.05)

Flow cytometry was used to quantify different immune cell populations that could facilitate QD transport from skin to the draining lymph nodes following a topical application using the experimental protocol described above (Fig. 1). The cell populations analyzed included MHCII (Major Histocompatibility Complex II), which is present on antigen-presenting dendritic cells; CD207 (anti-langerin) for LCs; F4/80, for mouse macrophages and CD11b (broad leukocyte marker), which is primarily expressed on granulocytes, monocytes/macrophages, dendritic cells, NK cells, and subsets of T and B cells. Results showed an overall increase for QD605+ events in the lymph nodes when applied on day 4 post-UVR and similar trends were present in all of the individual cell populations that were analyzed (Fig. 7, red squares). The number of MHCII+, CD207+ and Cd11b + events (Fig. 7a, b and c) were high in the lymph nodes on day 4, which is expected because dendritic cells migrate out of the skin post UVR exposure as described earlier (Fig. 5). The CD207+ events on day 4 (Fig. 7b) were significantly higher compared to control (*p* < 0.05), which is consistent with their migration from the skin to the draining lymph nodes post UV irradiation. We expected to measure fewer CD207 + QD605+ events on day 4, since most LCs (~70%) have migrated out of the skin at the time of QD skin application; therefore, fewer LCs would be present in skin to uptake QDs and transport them to the draining lymph nodes. Our results indicate however, that the CD207 + QD605+ cell numbers positively correlate with the increased TEWL barrier defect. Together this data suggests that non-cellular transport of QDs occurs via the lymphatics or the blood stream with subsequent QD dendritic cell association occurring in the lymph nodes.

In addition, F4/80 + QD605+ (*p* < 0.05) levels were higher on day 4 compared to all other treatment groups (Fig. 7d). This is consistent with studies that report UVB induces chemoattraction of macrophages to skin [47, 48]. In another study, it was observed that CD11b + macrophages infiltrate the skin after UVR exposure and were a potent source for IL-10 production which may contribute to the UVR induced immunosuppressive microenvironment [49]. While it is likely that QDs can associate with macrophages in the lymph node, it is plausible that in our model macrophages that invade the skin post UVR exposure could also uptake the QDs and then transport them to the lymph nodes.

## Discussion

The skin is an important exposure route to engineered nanomaterials, principally from topical application of nano-enabled cosmeceuticals and UVR protective lotions [9]. In a previous study, we had reported an increased presence of QD in the skin using tissue histology and Transmission Electron Microscopy (TEM) following a 360 mJ/cm^2^ UV exposure in hairless mice [19]. However, we had not established outside-in NP penetration trends in correlation to the TEWL values that were measured in UV exposed mice [19]. UV skin exposure leads to the production of photoproducts, and DNA damage that ultimately cause apoptosis and sunburn (erythema) [50]. The high TEWL measured due to UVR induced inside-out water loss has been attributed to the loss of epidermal calcium gradient, disorganized lipids in the stratum corneum and altered immune responses in the skin [51–53]. It is expected that an increase in the barrier defect would lead to greater penetration of topically applied NP through skin and their subsequent interaction with the local immune environment. Contrary to what is expected, using AAS elemental tissue analysis we find similar levels of QD presence in in both intact and 180 mJ/cm^2^ UVR treated skin (Fig. 3b). Since our study did not differentiate QD localization in the stratum corneum from viable epidermal and dermal tissues this result suggests that QD retention in skin (that which could not be wiped off) is independent of the evolving barrier defect post UVR when the mice are treated with a 180 mJ/cm^2^ dose. Enhanced NP penetration through barrier-damaged skin has been reported by many groups [19, 22, 54–56]. When the mice were irradiated with a 360 mJ/cm^2^ dose, there was a significant increase in Cd levels in the skin tissue day 4 post-irradiation compared to control. This outcome is consistent with our previous result where we observed a greater QD presence in the skin post-UVR (360 mJ/cm^2^) using TEM and histology and likely results from the increased barrier defect due to an erythematous UVR dose [19]. Retention of QD in control skin (no UVR) likely results possibly from collection in defects in the stratum corneum layers, hair follicles or skin furrows which do not change significantly with 180 mJ/cm^2^ UVR. Interestingly, we also observed no change in the level of Cd in draining skin lymph nodes due to differences in QD application time after UVR exposure. While the percent of Cd retained in the lymph nodes was low (0.024% of applied dose) compared to the total Cd dose, this suggests there was no difference in the ability of these specific QDs to penetrate skin after 180 mJ/cm^2^ UVR exposure. While the quantity of QDs to penetrate skin is low, more studies should be performed to characterize the penetration of nanoparticles through skin in both chronic exposure and multiple application experiments, since biomedical and cosmetic nanoparticles are normally applied often and used for extended periods of time. Although rodent skin is thinner and more permeable than human skin, such studies are useful and relevant for risk assessment calculations and defining worst case scenarios.

Beyond skin penetration, the potential of nanoparticles to interact with immune cells in the skin is of interest not only for potential toxicological concerns due to inadvertent nanoparticle skin exposure, but also for the design of nanoparticle based drug delivery systems. Flow cytometry and AAS lymph node data suggests that the QDs may be transported by two mechanisms; one through the lymphatics or the blood stream, and the other involving cell (dendritic cell and/or macrophage) mediated transport. The trends for MHCII+ and CD207+ dendritic cells (DCs) in the lymph nodes (Fig. 7) align with migration of these populations out of the skin due to the well-studied phenomenon of UVR-induced immunosuppression and the increased skin barrier effect on Day 4 post UVR exposure [40]. AAS data did not show a peak in Cd levels in the lymph node on Day 4 post UVR (Fig. 3c) suggesting the presence of non-cell associated QD transport. At this point it is unclear whether cell-associated QDs in the lymph node were transported from the skin by dendritic cells or that QD uptake occurred in the lymph nodes (Fig. 7). However, while UVR exposure doesn’t seem to induce increased QD skin penetration at a dose of 180 mJ/cm^2^, there is an increased immune cell and QD co-localization in the lymph nodes. Therefore, the effect of nanoparticles on skin immune modulation with and without UVR exposure should be explored further.

## Conclusion

In conclusion, we have identified that QDs accumulate in skin regardless of skin barrier status at the 180 mJ/cm^2^ dose, and these QDs can transport to the lymph nodes. However, the 360 mJ/cm^2^ dose leads to overt barrier dysfunction resulting in higher QD retention in the skin. Based on confocal images and flow cytometry data, we can also conclude that antigen-presenting cells interact with and take up QDs; however, the transport from the skin to the draining lymph node likely includes both active (cell uptake) and passive (blood or lymphatics) transport. Due to the interaction with immune cells, ongoing studies are examining the immunomodulatory effect of topically applied nanoparticles both in the skin and systemically. In the future, we also plan to examine the differences between single nanoparticle applications and doses split over multiple days or weeks to examine the differences in skin penetration and retention of NP, since most cosmeceuticals containing nanoparticles are applied daily and multiple times per day.

## Additional files

Additional file 1: Figure S1. Quantification of Cadmium (Cd) in the feces. **Figure S2**. Gating Strategy used to analyze flow cytometry data. **Figure S3**. Atomic Absorption Spectroscopy (AAS) analysis data from spleen (180 mJ/cm^2^ UVR dose). **Figure S5**. Intradermal injection of QDs was used as a positive control to develop the flow cytometry protocol. (DOCX 8297 kb)

## Abbreviations

AAS: Atomic absorption spectroscopy
Cd: Cadmium
CNT: Carbon nanotubes
CPD: Cyclobutane pyrimidine dimers
GSH: Glutathione
ICP-MS: Inductively coupled plasma mass spectrometry
LC: Langerhans cells
NP: Nanoparticle
PEG: Polyethylene glycol
QD: Quantum dot
ROS: Reactive oxygen species
TEWL: Transepidermal water loss
UVB: Ultraviolet Radiation B
UVR: Ultraviolet Radiation
