# Supplementary material for: In vivo quantification of quantum dot systemic transport in C57BL/6 hairless mice following skin application post-ultraviolet radiation
Source: Part Fibre Toxicol. 2017 Apr 14;14:12. doi: 10.1186/s12989-017-0191-7 (PMC5391571; doi:10.1186/s12989-017-0191-7)
Supplement: Supplementary file 2 — Quantification of Cadmium (Cd) in the feces. Figure S2. Gating Strategy used to analyze flow cytometry data. Figure S3. Atomic Absorption Spectroscopy (AAS) analysis data from spleen (180 mJ/cm2 UVR dose). Figure S5. Intradermal injection of QDs was used as a positive control to develop the flow cytometry protocol. (DOCX 8297 kb) [file 12989_2017_191_MOESM1_ESM.docx]

**Figure S1. Quantification of Cadmium (Cd) in the feces.**

To assess the potential for QD ingestion, AAS analysis of excreted QDs in the feces of the mice used in this study (UVR treated mice + QD with collar) was compared to untreated control mice (Control), mice exposed to QDs topically without a collar, and mice exposed to QDs via oral gavage. The graph represents the mean +/- standard error, N=4. The statistics are based on a Kruskal-Wallis test with post-hoc Wilcoxon analysis. *p<0.05, significant with respect to Control. #p<0.05, Oral gavage significant with respect to all UVR + QD treated groups. $p<0.05, No collar QD treated group significant with respect to all UVR + QD treated groups.


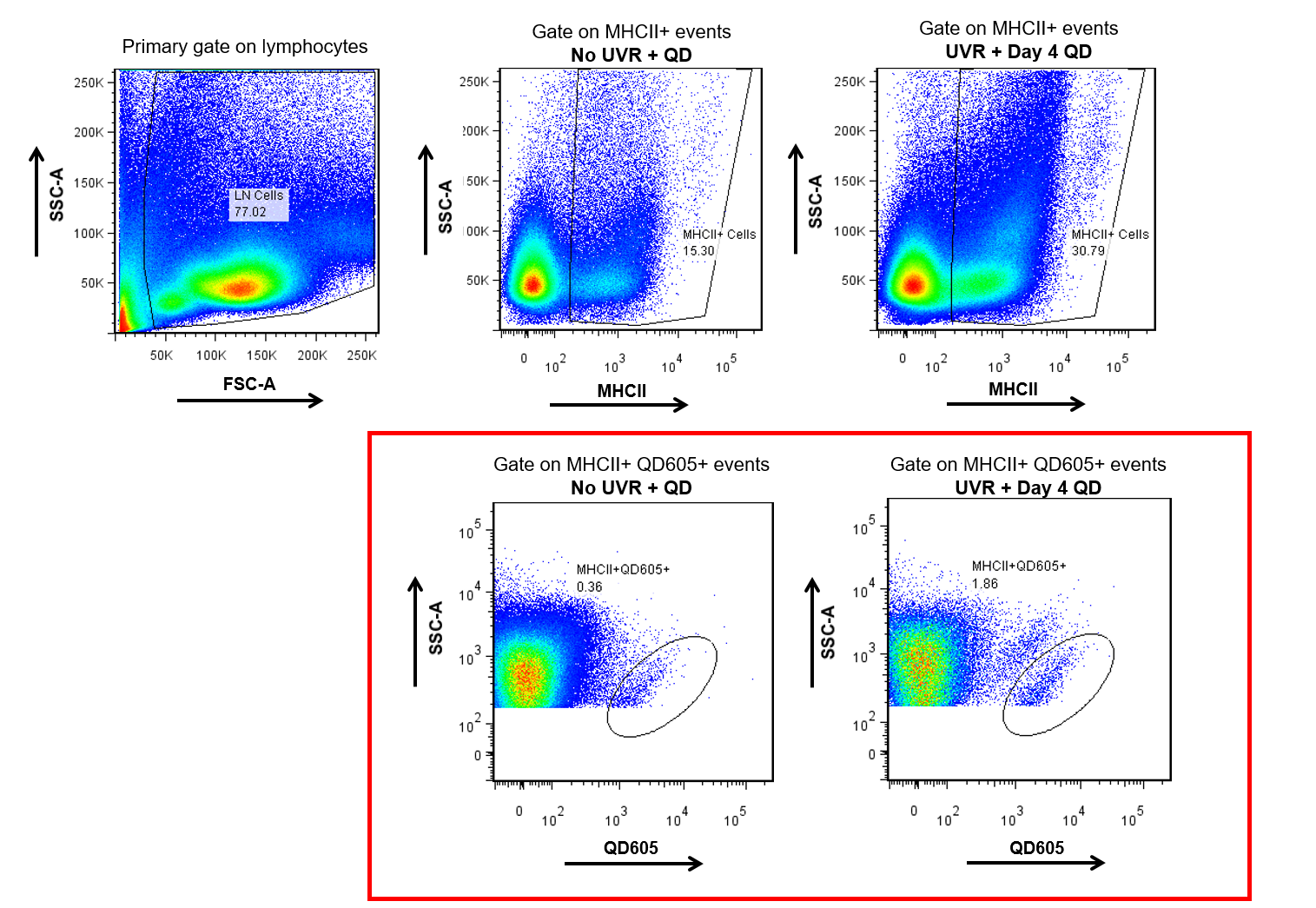


**E**

**D**

**C**

**B**

**A**

**Figure S2. Gating Strategy used to analyze flow cytometry data .**

This is a representative example of the gating strategy used to quantify different populations using flow cytometry. Beads were used for single stain controls to set the fluorophore voltages and calculate the compensation matrix on the flow cytometer. Approximately 10^6^ events were collected for each sample. However, for samples where total number of events were <10^6^, the data was normalized to 10^6^ events. The primary gate was drawn on all collected events to include lymphocytes in the analysis (77.02%) (A). The lymphocytes were next gated on MHCII+ events (antigen presenting cells) in the No UVR + QD group (15.30%) (B) and UVR + Day 4 QD group (30.79%) (C). The next gate was drawn on the MHCII+ events (B and C), shown in the red box (D and E). These events were double positive for MHCII+ QD605+ (antigen presenting cells co-localized with QD). The same gating strategy was repeated for other populations analyzed in this study. These scatter plots show the % positive events for each gate. FlowJo also gives the number of cells positive for each gated event, these cell numbers have been plotted in Figure 6 (normalized to a total of 10^6^ collected events per group).


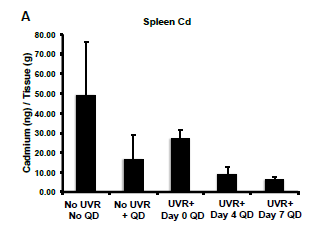


**Figure S3. Atomic Absorption Spectroscopy (AAS) analysis data from spleen**

**(180 mJ/cm^2^ UVR dose).**

The concentration of cadmium (ng/g tissue) found in the spleen. The graphs represent the mean +/- standard error (SEM), N=4. The results were analyzed using a one-way ANOVA and no significant differences were found between different treatment groups and the No UVR No QD control.

**Figure S5**. **Intradermal injection of QDs was used as a positive control to develop the flow cytometry protocol.** 24 hours post injection, lymph nodes were harvested and the skin was stained for LCs, scale bar=50 µm (A). The Pearson’s coefficient for co-localization in the skin, r=0.829. QD605+ clusters were also detected in the axillary lymph nodes, scale bar=100 µm (B), which was confirmed using flow cytometry analysis (C).
